# Extracellular Vesicles from Immune Cells: A Biomedical Perspective

**DOI:** 10.3390/ijms241813775

**Published:** 2023-09-07

**Authors:** María José Moya-Guzmán, Javiera de Solminihac, Cristina Padilla, Carolina Rojas, Camila Pinto, Tomás Himmel, Karina Pino-Lagos

**Affiliations:** Centro de Investigación e Innovación Biomédica, Facultad de Medicina, Universidad de los Andes, Av. Plaza 2501, Las Condes, Santiago 755000, Chile

**Keywords:** extracellular vesicles, exosomes, immune cells, immune regulation, immunity

## Abstract

Research on the role of extracellular vesicles (sEV) in physiology has demonstrated their undoubted importance in processes such as the transportation of molecules with significance for cell metabolism, cell communication, and the regulation of mechanisms such as cell differentiation, inflammation, and immunity. Although the role of EVs in the immune response is actively investigated, there is little literature revising, in a comprehensive manner, the role of small EVs produced by immune cells. Here, we present a review of studies reporting the release of sEV by different types of leukocytes and the implications of such observations on cellular homeostasis. We also discuss the function of immune cell-derived sEV and their relationship with pathological states, highlighting their potential application in the biomedical field.

## 1. Introduction

The immune system is considered a complex collection of molecules, cells, and organs that function to eradicate infections, conferring long-lasting protection. In addition to microbial antigen recognition, immune cells can identify self-antigens under healthy and disease conditions. In the first scenario, we refer to immune tolerance, whereas in the second one, we denote autoimmunity. Regarding the timing involved in triggering immune activation, we could mention a fast, general response coined innate immunity, in which cells such as neutrophils, eosinophils, and macrophages are the main players. In this reaction, no antigen specificity is involved. On the other hand, antigen-presenting cells that capture, process, and present antigens to CD4+ T cells, CD8+ T cells, or B cells elicit an efficient but slower response that is specific and can generate memory. This type of reaction is called adaptive or acquired immunity. In these two processes, a controlled or regulatory event occurs to prevent self-destruction, and the main cells participating in this mechanism are CD4+ T regulatory cells (Tregs), although other cell types have shown suppressive function as well (i.e., tolerogenic dendritic cells (DCs), B regulatory cells (Bregs), myeloid-derived suppressor cells (MDSC), and macrophages with anti-inflammatory function (or M2-type)). Most of the mentioned cell types have been shown to secrete small extracellular vesicles (sEV) as a way to exert their immune activity.

The discovery of the “vesicle transport system” brought to light the existence of different types of microvesicles, which can be grouped according to their origin, size, or structure. For instance, larger extracellular vesicles (EV), such as apoptotic bodies, have a size of 800–5000 nm; medium-size EV, which originated from membrane shedding, have a size of 50–3000 nm; and lastly, small EV (sEV) or exosomes, which correspond to multivesicular body-derived EV, have a small size of 50–150 nm. To date, many reports have indicated that any cell type, of hematopoietic or non-hematopoietic origin, can secrete sEV, including immune cells. sEV originates from the multivesicular bodies/late-endosome and is released when they fuse with the plasma membrane. They play an important role in intercellular communication and may act as “packages” for transferring proteins, lipids, mRNA, and cytokines from one cell to another, modulating the receptor cell. They are composed of a phospholipid bilayer membrane containing membrane-associated proteins, some of which serve as exosome markers: Alix, TSG-101, CD63, CD9, CD53, and others [1]. Furthermore, the cargo of sEV can be composed of products from the donor cell, having as major components mRNA, miRNA, DNA, lipids, and proteins [2]. Because of the profound relevance that these molecules play in the body and considering the unquestionable importance of the immune system, we sought to revise the literature regarding the production of sEV by immune cells and its consequences for the organism. 

It is important to note that the searched literature was based on terms such as “small extracellular vesicles”, “extracellular vesicles”, and “exosomes”, and the studies included here were selected because of their relevance, the vesicles obtained (technique), and their characterization. Thus, if the vesicles were isolated by ultracentrifugation, CD63-based kits, or size exclusion chromatography and the authors described a size between 50 and 150 nm plus the presence of markers such as those mentioned above, we used the term sEV.

In the following sections, we present several studies subdivided into three major groups given by the cell types studied: first line of defense (which includes neutrophils, eosinophils, basophils, mast cells, and NK cells), antigen-presenting cells (including dendritic cells, macrophages, myeloid-derived suppressor cells, and B cells), and T cells (CD8+ T cells, CD4+ T cells, T regulatory cells, and follicular helper T cells).

## 2. Cells in the First Line of Defense

### 2.1. Neutrophils

Generated in the bone marrow, neutrophils are the most abundant leukocytes in human peripheral blood, reaching between 50 and 70% of total cells. They are part of the first line of defense against pathogens but can also respond to other inflammatory scenarios, such as cancer or obesity. Mature neutrophils exert their mechanism of action through various functions, including phagocytosis, the release of granules containing metalloproteinases and antimicrobial enzymes, and the formation of neutrophil extracellular traps (NETs) [3].

Neutrophils secrete extracellular vesicles (EVs), but most studies do not refer to small EV (sEV) because, upon characterization, neutrophil-derived EVs show a wide size range comprising particles of 100 to 500 nm (or bigger), which has facilitated their analysis by flow cytometry. These studies indicated that molecules such as CD66b, CD11b, CD18, and MPO are present in their membrane [4]. In 2018, Álvarez-Jiménez et al. characterized the particles released by un-stimulated, PMA-stimulated, bacterial protein-stimulated, and intracellular (*Mycobacterium tuberculosis*, Mtb) bacteria-stimulated neutrophils, showing that the size of the secreted vesicles depends on the stimuli. For instance, un-stimulated and bacterial protein-stimulated human neutrophils produced vesicles with 100–200 nm in size, whereas PMA-activated and Mtb-stimulated neutrophils released vesicles with 100–300 nm and >200 nm in size, respectively. Among these 4 different types of vesicles, those obtained from Mtb-stimulated neutrophils induced the secretion of TNF-α, IL-6, and IL-10 and the expression of CD86 on macrophages. Mtb-infected macrophages treated with these vesicles provoked a clearance of intracellular Mtb, possibly due to the production of nitric oxide and the activation of autophagy [5]. Other studies in which EVs enriched from mouse and human neutrophil cultures were characterized showed that murine vesicles are smaller than human ones (fractions of 10–15 nm and 100–200 nm, compared to ~100 nm and 200–700 nm, respectively). Also, they demonstrated that human EVs with antibacterial properties correspond to medium-size EVs (200–700 nm) and not to sEV (<200 nm). Although they did not test whether the bigger EVs from mice had the same activity as the human counterpart, both fractions were sensitive to Triton X-100 detergent treatment, indicating their vesicular nature [6]. Another recurrent variable in neutrophil-derived EVs studies is the stimulation of the cells with bacteria (or bacterial components) or the use of pro-apoptotic neutrophils, but, once again, the EVs fraction investigated does not correspond to sEV [4]. Last, Bonifay et al. performed a robust screening for molecules that could be used as neutrophil-derived EV markers. The authors used human neutrophils and enriched for EVs using size-exclusion chromatography (SEC) and demonstrated that the EVs had an approximate size of ~190 nm. Using flow cytometry, they screened for 54 molecules, 8 of which were neutrophil-specific (CD15, CD66b, CD66c, CD16, CD13, CD32, lactoferrin, and TIA-1). This work offers a new strategy to identify neutrophil-derived EVs (under healthy and pathological conditions) and to search for potential biomarkers [7].

### 2.2. Eosinophils

Eosinophils develop in the bone marrow; they can be found in the circulation and in tissues. In the periphery, eosinophils correspond to up to 6% of human blood leukocytes. They possess granules containing a diverse type of mediator and play a fundamental role in allergic reactions, Th2-type immune responses, gastric/intestinal inflammatory diseases, and cancers, among others. For a comprehensive description of eosinophil biology, we recommend reading Gigon et al.’s review [8]. In 2008, Neves et al. reported that vesicles produced by eosinophils contained proteins currently used as sEV markers (CD63 and LAMP-2, [9]) and other molecules that can be released to exert function, such as cytokines. Although they do not refer to or discuss the possibility that these granules correspond to or contain sEV [10], another group led by del Pozo investigated this opportunity. In the study, they show that IFN-γ or eotaxin-stimulated human eosinophils secrete vesicles with an average size of 162 nm, the presence of CD63, CD9, and Alix, and a corresponding nanovesicle shape as seen on transmission electron microscopy analysis, indicating features of sEV. The same type of vesicles was obtained from asthmatic patients, but in greater numbers, suggesting a potential role in this disease [11]. Later, the same research team demonstrated that exosomes isolated from healthy and asthma patients affect eosinophil phenotype and function, in addition to exerting an impact on epithelial and muscle cell lines viability and gene expression, only when eosinophil-derived exosomes from asthma patients are used [12,13]. In other words, “asthma exosomes” carry messages of the disease, which affects not only eosinophils but other cell types present in the lungs. It is important to note that none of these publications use the term “small Extracellular Vesicles”.

### 2.3. Basophils

Basophils are granulocytic cells that originate in the bone marrow as well and correspond to a scarce cell population. Human basophils were first identified and described more than 140 years ago, whereas mouse basophils were characterized in 1982 by microscopic analysis [14]. In general, basophils are found in the peripheral blood and migrate to tissues under inflammatory conditions. Basophils and mast cells were for many years considered the same cell type due to their morphological similarities and their unique capacity to produce and release histamine. Basophils play an important role in allergic reactions due to the expression of FcεR, which can bind IgE and trigger the release of granules/vesicle content; they can also produce cytokines such as IL-4 and IL-13, stimulating the differentiation of Th2 cells; however, basophils may also participate in tissue repair and in other pathological conditions such as cancer [15].

The observation of vesicle production by basophils came from early studies investigating the degranulation process [16]. Following research using macroscopic tools, it was described the kinetics and morphological changes of basophil plasmatic membrane (membrane folding, irregularities, release/reincorporation of material) upon cell activation, discussing the biologic relevance of those vesicles containing (or not) granules [17]. Even though some publications refer to “extracellular vesicles”, there are no reports on sEV isolated from basophils under any condition.

### 2.4. Mast Cells

Mast cells originate in the bone marrow. They play a major role in allergy responses through the release of several molecules present in their granules. As with other immune cells, mast cells also release sEV. In this regard, in 2001, the group of Skokos and colleagues prepared sEV obtained from murine BM-derived mast cells and from cell lines. They found that mast cells secreting sEV with an average size of 60–100 nm, which contain molecules such as MHC-II, CD86, LFA-1, and ICAM-1, induced the activation/proliferation of splenocytes in vitro and the production of the pro-inflammatory cytokines IL-2 and IFN-γ, which favor Th1-type responses. These findings were recapitulated in mice, which received mast cell-derived sEV via intraperitoneal injection, resulting in lymphocyte activation since the production of IL-2 and IFN-γ was up-regulated in the mesenteric lymph nodes of treated animals. Interestingly, the production of these modulating sEV was dependent on IL-4, since mast cells produced in the absence of this cytokine were unable to activate lymphocytes [18]. A follow-up study by the same team evaluated the immunogenic properties of murine mast cell-derived sEV loaded with exogenous antigens (transferrin [Tf], bovine serum albumin [BSA], and ovalbumin, [OVA]) and the ability to present antigen to T cells. By binding Tf or BSA to syngeneic mast cell-derived sEV and treating mice with them, the authors observed that sEV elicited a strong humoral response given by high titers of IgG1 and IgG2a. These findings were complemented with DCs data, in which DCs treated with mast cell-derived sEV and not from other origins became mature (high expression of MHC-II, CD40, CD80, and CD86) and released elevated amounts of IL-12. The results demonstrated mast cell-derived sEV’s ability to prime the immune system by serving as a source of antigen and acting as an adjuvant (presumably due to the presence of Hsp60 and Hsp70). In vivo, the authors administered mast cell-derived sEV bound to OVA into mice, and after purifying DCs from lymph nodes, they demonstrated that only DCs from sEV + OVA-treated animals (and not from PBS or OVA alone-treated groups) triggered IL-2 secretion from cell lines in vitro, confirming the ability of sEV as a manner of Ag delivery with an immunostimulatory effect [19]. As with sEV obtained from other cell types, those isolated from mast cells also contain RNA, miRNA, and long noncoding RNA, which are transferred to other mast cells or cell types [20,21]. In this regard, a study investigating the inflammatory role of mast cell-derived sEV showed that miRNA-21 was present in these sEVs to mediate asthma. The report demonstrated that the mouse airway epithelial cell line (MIC-iCell-a006) contained elevated miRNA-21 after mast cell-derived sEV, and the use of an inhibitor against this miRNA showed increased cell activity, alleviating oxidative stress and inflammation. In vivo, using a mouse model of asthma, the authors demonstrated that miRNA-21 increased in diseased animals, which was associated with down-regulation of antioxidative enzymes and up-regulation of inflammatory parameters. The activity of miRNA-21 was linked to the Wnt/β-catenin pathway [22].

### 2.5. Natural Killer Cells

**Figure 1:**
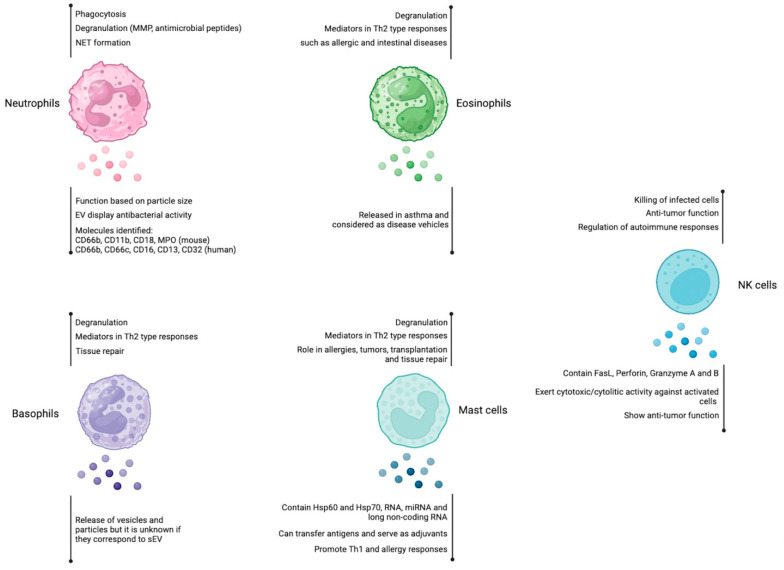
Role of extracellular vesicles derived from neutrophils, eosinophils, basophils, mast cells, and NK cells. Neutrophils, eosinophils, basophils, and mast cells are immune cells that participate mainly in Th2-mediated responses, whereas NK cells display cytotoxic activity. Among their effector mechanisms, they use degranulation to release different mediators. The involvement of EVs in the immune response is indicated for each cell type (below the vesicle´s shape). Figure created at BioRender.com.

NK cell-derived sEV has been studied for at least a decade, resulting in the use of CD56 as a marker to identify sEV of NK cell origin [23]. According to Fais et al.’s work, sEV obtained from healthy human donors contains CD56 in addition to Perforin, FasL, NKp46, and NKG2D and shows cytotoxic activity against tumor cell lines and activated cells, suggesting a beneficial role in preventing tumorigenesis. Similar findings were also reported in another study in which human NK cell-derived sEV showed cytolytic activity against hematological cancers such as leukemia and lymphoma but not against solid tumors. Interestingly, the authors report that NK cell-derived sEV cytotoxicity can be seen either from activated or resting human NK cells as a sEV source and that cytotoxicity does not depend on FasL but on perforin [24]. Because of the anti-tumoral effect seen on NK cell-derived sEV, a study was intended to develop a protocol to produce (in a G-Rex bioreactor) and isolate (using PEG8000 to precipitate) large amounts of human NK cell-derived sEV with the purpose of clinical application. The authors used “artificial” APC that displays IL-21 on their cell membrane (K562 Clone 9.mbIL21 cells) to strongly activate NK cells. Next, they isolated NK cell-derived sEV and proved sEV identity using NTA, transmission electron microscopy (TEM), and Western blot analysis. Their findings indicated that NK cell-derived sEV produced in large quantities contains Granzyme A and Granzyme B, as well as Perforin, and demonstrated that sEV cytotoxic activity against tumor cells is mediated by the caspase pathway [25]. In the same vein, research performed by Zhu et al. utilizing in vitro and in vivo bioluminescence experiments found that murine NK cell-derived sEV contained Perforin and FasL and was able to inhibit tumor growth using a murine melanoma model (using B16F10 murine cells). Importantly, NK-cell-derived sEV did not show adverse effects on healthy cells [26]. In general terms, most of the studies on NK cell-derived sEV relate to cytotoxicity, directing their use as antitumor agents. Figure 1 summarizes the main characteristics and properties of sEV obtained from neutrophils, eosinophils, basophils, mast cells, and NK cells.

## 3. Antigen Presenting Cells

### 3.1. Dendritic Cells

Dendritic cells (DCs) are one of the main orchestrators of the immune response, serving as a bridge between innate and adaptive immunity. DCs are responsible for sensing changes in the environment, such as signals of cell damage and the presence of pathogens, and for exerting an effector function to eradicate the insult. DCs are very heterogeneous, but all originate from a common DC precursor that gives rise to plasmacytoid DCs (pDCs) and conventional DCs (cDCs). pDCs are mainly involved in IFN type I production in response to pathogens, while cDCs act mainly as antigen-presenting cells (APCs) [27]. Activated cDCs go through a maturation process to become “professional” APCs, experiencing morphological changes, increased expression of surface molecules such as the antigen-presenting major histocompatibility complex (MHC) I and II, co-stimulatory molecules CD86, CD80, and CD40, and increased secretion of different cytokines that will define a specific T cell response [28].

Some of the first characterizations of sEV from murine bone marrow-derived DCs (BM-DCs) were reported by Zitvogel et al., who described that sEV contained functional MHC-I and MHC-II as well as the co-stimulatory molecule CD86 and the transferrin receptor (TfR) [29]. When these DCs were pulsed with autologous acid-eluted tumor peptides (from P825 and TS/A tumors) and then administered as a single injection into tumor-bearing mice, the treatment promoted significant retardation of tumor growth in most animals. DCs-derived sEV administration was more effective than using DCs as a therapy [29]. To further understand possible mechanisms related to the effect of this sEV on tumor suppression, the murine DC cell line D1 was used as a model to characterize the derived sEV protein composition [30]. The authors determined that the production of sEV by DCs is developmentally regulated, being more effective in immature than in mature DCs. Also, these sEV accumulated a unique subset of cellular proteins that included the membrane proteins MFG-E8, Mac-1, and CD9 and some cytosolic proteins such as hsc73 and Annexin II. Given that Hsc73 has been described as a potent inducer of antitumor immune responses in vivo, the authors proposed that this protein could also be involved in the sEV antitumor effects [30]. Later studies using murine DCs and *Drosophila*-derived cells transfected with murine molecules demonstrated the direct effect that DCs-derived exosomes (100 nm) exert on CD8+ T cells in vitro, showing that these vesicles can be recognized by murine naïve CD8+ T cells in the absence of viable APCs and can exert an immunogenic response when ICAM-1 and CD80 are present [31]. Unlike naïve CD8+ T cells, in vitro studies using murine naïve CD4+ T cells suggested that DCs-derived sEV did not induce antigen-dependent T cell stimulation unless mature DCs were also present, indicating that sEV promotes the exchange of functional peptide-MHC complexes between DCs. The authors proposed that this mechanism could increase the number of DCs bearing a particular peptide, thus amplifying the initiation of primary adaptive immune responses [32]. Additionally, this group has described that even though there is less production of sEV from murine DCs in the mature state (LPS-induced), the protein composition of these vesicles is different from sEV secreted from immature DCs. This is a reflection of changes in DCs protein expression during maturation, with an increase in sEV of MHC-II, CD86, and ICAM-1 and a decrease in MFG-E8, which is abundant in immature DCs. However, they maintained the levels of exosomal proteins such as Hsc70, Annexin II, Clathrin, or CD9 and their overall morphology [33,34]. When the ability of both types of sEV to activate T cells was compared, sEV from mature DCs was more effective in vitro [33] and could transfer to inefficient APCs, such as B cells, the ability of DCs to activate naive T cells. This observation was not found when B cells were treated with sEV obtained from immature DCs [34]. Also, the efficiency of sEV produced from in vitro matured DCs to induce T cell activation was demonstrated in vivo using skin graft assays, as was the requirement for MHC class II and ICAM-1 molecules to execute this response [34]. Among other DC-derived sEV functions is their ability to bind LPS and then activate other DCs in a bystander manner. This activation increased murine DCs expression of transmembrane TNF (tmTNF), secretion of cytokines, and promotion of NK cells to augment IFN-γ release, boosting the host immune system [35]. sEV are also able to contain tmTNF; in fact, it has been described that sEV from murine mature DCs is involved in the acceleration of endothelial inflammation and atherosclerosis development. The tmTNF present in these sEV was able to activate HUVEC cells via NF-kB, and in vivo studies showed that these sEV could be taken up by aortic endothelial cells and induce inflammation and atherosclerosis in mice [36]. Regarding DCs-derived sEV cargo, Montecarlo et al. described that murine bone marrow-derived DCs produced sEV with different miRNA compositions depending on their maturation state (immature and mature). They reported that of the 139 miRNAs in sEV from immature and mature DCs, five were only present in immature sEV, and 58 were exclusively present in mature sEV. These miRNAs have been linked to regulating critical functions in DCs. The authors demonstrated that these sEV were internalized, hemifused, and/or fused with other target DCs, proposing an additional mechanism of miRNA transfer between DCs [37].

Experiments using different human DC-derived sEV showed that sEV and large EVs (lEVs) secreted from immature DCs produced different Th responses, where lEVs favored the secretion of Th2 cytokines and sEVsfavored the secretion of Th1 cytokines. However, when DCs were matured with IFNs, all sEV produced were able to promote a Th1 response, regardless of their size [38]. Furthermore, it has been described, using in vitro assays, that sEV from human naïve DCs can be internalized by mesenchymal stromal cells (MSC) and promote their recruitment and migration, which was linked to the presence of the chemokine Osteopontin and the Matrix Metalloproteinase-9 (MMP-9) present in the sEV [39].

### 3.2. Macrophages

Macrophages are mononuclear cells that are very important for the innate and adaptive immune systems. In general terms, they can be classified into two major groups: M1, a pro-inflammatory activated cell, and M2, which is an anti-inflammatory cell [40,41]. In the inflammatory context of liver fibrosis produced after schistosomiasis, a study reported that sEV produced by murine macrophages could serve as a communication mechanism between these cells and hepatic stellate cells. In the study, the authors demonstrated that schistosome egg antigen-activated macrophages released sEV containing miR-33, inducing TGF-β1 signaling on hepatic stellate cells, which causes their activation and progression of the disease [42]. An additional study focused on protein tyrosine-nonreceptor phosphatase type 1 (Ptpn1), a key protein for the polarization of macrophages toward the pro-inflammatory phenotype. These authors observed that decreasing the expression of Ptpn1, in the context of intestinal inflammation, caused murine macrophages to become anti-inflammatory and release sEV without Ptpn1, restoring homeostasis of the intestinal tissue. Therefore, in the context of chronic inflammation, the inhibition of Ptpn1 could be beneficial [43]. Another study showing the importance of macrophages in old female mice was published in 2022, which reported that both M1 and M2 polarized macrophage-derived EV regulated primordial follicle activation. In the report, M1-EV increased AKT phosphorylation in primordial follicles, whereas M2-EV decreased it. This observation was attributed to the presence of miR-107 on M1-EV and miR-99a-5p on M2-EV. Interestingly, the treatment of M2-EV containing miR-99a-5p improved ovarian function in old female mice, observing an increase in the number of follicles and a reduction in follicular atresia. In this study, the authors only used NTA to characterize the size and concentration of the vesicles [44]. Another study found that sEV from macrophages induce inflammation in the microenvironment of artificial prostheses in mice. These macrophage-derived sEV contained miRNA-3470b, a microRNA present in inflammatory osteolysis, in addition to TGF-β, IL-6, and IL-1β, which are factors known to promote prosthetic rejection [45]. In the scenarios described above, the new knowledge on macrophage-derived sEV permits the development of new strategies to prevent or control inflammatory conditions.

As is known, the tumor microenvironment shows dual functions of the immune cells, where tumor-associated macrophages (TAM) play an important role against lung cancer metastasis. Recently, a direct relationship was observed between macrophage-derived sEV containing large amounts of TNF-α and the expression of the pulmonary surfactant protein (SP) in epithelial cells from human samples [46], indicating a dysfunction in epithelial cells from acute lung injury. In the same context of inflammation, another publication studied sEV produced by M1-polarized macrophages with elevated glucose levels isolated from patients with diabetic foot ulcers. The authors found that M1-derived sEV had a large load of iNOS in addition to miR-503’s presence, which downregulated the expression of Igf1r (a gene that plays a protective role in epithelial regeneration). These results demonstrated that M1-derived sEV induced dysfunction in HUVEC cells and had a negative effect on wound healing in diabetic mice [47]. Another promising study revealed that rat CD68+ cardiac macrophage-derived sEV may help with heart inflammation and repair after myocardial infarction, where sEV could induce a decrease in the population of CCR2+ (pro-inflammatory) macrophages [48], thus showing one of the many uses of sEV in biomedicine. Using the human NSCLC cell line and Balb/c mice, Huang et al. demonstrated that TAMs help the migration of cancer cells through the release of sEV containing high levels of αV and β3 integrins. In addition, they observed that patients with a worse prognosis had higher levels of these integrins compared to non-metastatic patients. Therefore, blocking this integrin present in the TAM-derived sEV could be a very good mechanism of action for future therapies [49].

However, macrophages, being cells of the immune system, depending on the context, can also help to attenuate inflammation. In a study that followed a microRNA noted for its “ability” to relieve inflammatory pain in a mouse model, it was found to be present in macrophage-derived sEV. The miR-23a-3p was produced by M2 type macrophages and targeted via sEV to microglia, where it targeted USP5 for regulating the HDAC2/NRF2 complex, leading to a decrease in inflammatory pain [50]. In the same context, a recent article published suggested that sEV derived from macrophages transfected with the lysosomal enzyme tripeptidyl peptidase-1 (TPP1), an enzyme used as a target for treating Batten disease (BD, a pathology that affects the nervous system in children), could be used as an alternative cure for this pathology. Being a disease that affects the nervous system, the administration of a localized drug is not easy, and in this case, the authors were able to administer TPP1 effectively and cumulatively through sEV via intrathecal and intraperitoneal routes in the BD mouse model, observing a significant therapeutic effect [51]. These results demonstrate the positive therapeutic effects that can be harnessed from macrophages, cells that cross the BBB and could deliver these sEV loaded with therapeutic agents.

### 3.3. Myeloid-Derived Suppressor Cells

Myeloid-derived suppressor cells (MDSC) are cells of myeloid origin; they express Gr1 and CD11b, present antigens, and display regulatory function. They have been widely studied in tumor models where they promote tumor growth by suppressing anti-tumor immunity through the expression and secretion of anti-inflammatory molecules (cell membrane ligands and cytokines) and the activity of Arginase-1 (Arg-1) and iNOS enzymes, although their role in different pathological contexts other than tumors has been described as well [52].

The first study referring to “exosomes” released by MDSC demonstrated that they secrete 25–30 nm size particles that bear a protein cargo that varies upon inflammation. MDSC were isolated from the blood of mice injected with mammary carcinoma cells or with IL-1β-transfected mammary carcinoma cells as an inflammatory stimulus. Proteomic analysis showed that 400 proteins are present in exosomes obtained from tumor mice +/− IL-1β, highlighting S100A8 and A9 as chemotactic molecules contributing to MDSC suppressive function. Furthermore, MDSC-derived exosomes could switch macrophage polarization to favor the pro-tumor M2-type [53]. Using the same tumor model, another report indicated that murine MDSC-derived sEV contained miR-126a, which mediates tumor progression and angiogenesis, and RNAs coding for molecules linked to MDSC suppression [54,55]. In the context of intestinal inflammation, administration of murine MDSC-derived sEV prevented the development of colitis by down-regulating inflammatory cytokine release into the blood, Th1 cell generation, and Tregs development. Interestingly, the protective role of MDSC-derived sEV depended on Arg-1 function [56]. In a mouse model of alopecia, MDSC-derived sEV was shown to prevent hair loss by promoting Tregs and decreasing Th1 cell frequencies. In this study, Foxp3 and Arg-1 mRNAs were upregulated upon MDSC-derived sEV treatment [57]. In an alternative experimental model, a study showed that the administration of MDSC-derived sEV into arthritic mice controlled the differentiation of Th1 and Th17 cells. The authors discussed the potential role of miR-29a-3p and miR-93-5p, both present on MDSC-derived sEV, as agents to control pro-inflammatory T cell responses [58]. Furthermore, the same research group reported that MDSC secrete sEV, and their administration into arthritic mice prevents disease progression [59]. A couple of more recent studies have shown the beneficial effect of MDSC-derived sEV in experimental mouse models of autoimmune hepatitis and Sjögren’s syndrome, where sEV is pivotal to controlling inflammation driven by mitochondrial damage and B cell function, respectively [60,61].

### 3.4. B Cells

B cells comprise a key component of the adaptive arm of the immune system. They are responsible for the short- and long-term generation of humoral antibody responses, participate in antigen presentation, and modulate effector immune cell responses. [62]. B-cell-derived sEV has been proposed to play a role in multiple biological processes associated with B-cell development, maturation, and functionality. Like on T cells, B cell-derived sEV secretion can be stimulated by activation and/or inflammatory signals [63], such as TCR:MHC-II interaction and CD40/CD40L or interleukins stimulation [64].

The first mechanism associated with sEV obtained from B cells described MHC-II molecules’ secretion within sEV, which were released from the specialized APCs late endocytic compartment, MIICs (MHC class II-enriched compartment). Exosome-bound MHC-II from both human and murine B cells was present in a compact, peptide-bound conformation capable of inducing antigen-specific MHC-II-restricted T cell responses [65]. On the other hand, sEV has also been suggested to participate in B cell development within the BM. Murine primary BM-derived B cells and a murine pre-B cell lymphoma cell line (WEHI-231) were shown to release CD24+ plasma membrane-derived sEV upon CD24 engagement [66]. CD24 (also known as heat-stable antigen) is a surface protein that promotes apoptosis, and its expression is detectable in the early stages of B cell development and suffers a gradual decline once B cells become mature and exit the BM [67]. CD24+ sEV exchange between immature B cell populations was proposed to participate in B cell development by acting on their selection and differentiation processes. Furthermore, CD24 or B cell receptor (BCR) stimulation on a B cell lymphoma cell line triggered the secretion of sEV that carried functional BCR and CD24 to recipient B cells. These additional “transferred” BCRs permitted recipient B cells to respond to novel antigen stimulation. Additionally, these cells were endowed with higher sensitivity to CD24-mediated apoptosis. It is important to note that this mechanism explained only a small fraction of BCR gain mechanisms, as only 5–20% of B cells gained new BCR by acquiring sEV [66].

Finally, in the biomedical context, human B cell-derived sEV has also been studied as a useful tool for disease prognosis assessment. CD19+ sEV that carried CD39 and CD73 ectoenzymes was shown to sequentially hydrolyze ATP from tumor cells into adenosine, a highly immune-suppressive molecule that inhibits T cell activation, impairing post-chemotherapeutic CD8+ T cell responses. Patients with lower levels of serum CD19+ sEV had a better prognosis after chemotherapy. Thus, it was proposed that strategies that could decrease CD19+ sEV secretion (such as Rab27a siRNA, tested on humanized immunocompromised mice) showed a high potential for improving anti-tumor chemotherapy efficacy [68].

**Figure 2:**
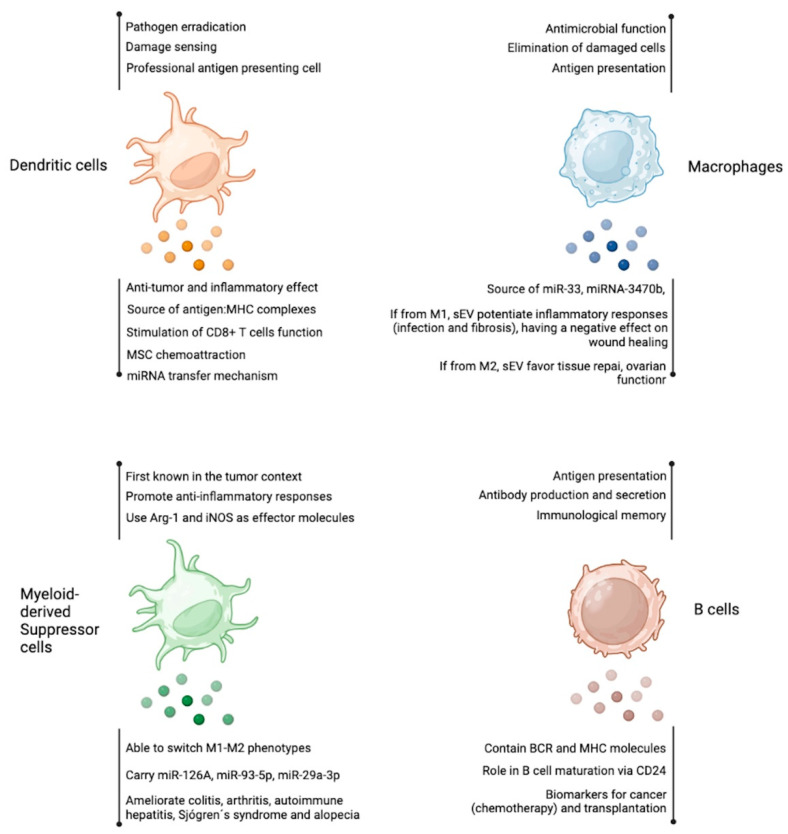
Antigen presenting cells-derived extracellular vesicles. As other cell types, dendritic cells, macrophages, myeloid-derived suppressor cells, and B cells produce EV with several reported functions. Among them, the transfer of miRNA and the source of antigens are the most studied features of these EVs. Figure created at BioRender.com.

More recently, Cucchiari et al. found that the evaluation of human B cell-derived sEV in living-donor kidney transplant recipients undergoing pre-transplantation desensitization for human leukocyte antigen (HLA)-incompatibility could indicate B cell residual activity after desensitization. Circulating CD19+ HLA-II+ sEV suffered a significant drop following desensitization, which reflected the reduction of CD19+ B cells in lymph nodes (with no differences in circulating B cells). Also, patients who suffered antibody-mediated rejection developed a significant rebound in circulating B-cell-derived sEV, suggesting re-proliferation or differentiation of alloreactive B cells. Based on these results, it was proposed that B cell-derived sEV kinetics and function assessment in clinical practice could reveal humoral alloreactivity during transplant rejection or, potentially, in other settings [69]. Figure 2 shows in a concise manner the findings discussed above.

## 4. Cell Types from the CD8+ T Cell and CD4+ T Cell Compartment

### 4.1. CD8+ T Cells

Generated in the thymus, CD8+ T cells contribute to the elimination of intracellular pathogens like bacteria, viruses, and parasites, as well as tumor cells. These cells are activated when the T cell receptor (TCR) recognizes antigens presented by MHC-I on the APC surface [70]. Effector CD8+ T cells help to eliminate pathogens through cytokine secretion and direct killing of infected cells, whereas memory CD8+ T cells provide enhanced, long-lasting protection from reinfection [71]. In the context of EV, Peters et al. demonstrated in 1991 that exosomes derived from murine and human CD8+ T cells contain granzyme and perforin, which are exocytosed during specific interactions with target cells, resulting in the killing of these target cells [72]. Furthermore, these cytotoxic T cells can be stimulated with IL-12 to secrete exosomes, as shown by Li et al. [73]. In that study, IL-12 stimulation affected the size of vesicles derived from murine T cells, and when activated, the resulting vesicles contained exosome markers such as Alix, CD81, TSG101, Flotillin-1, and activation-related molecules from cytotoxic T cells, such as STAT3 and STAT5B. It was also demonstrated that IL-12-stimulated exosomes from cytotoxic T cells activate CD8+ T cells in the absence of antigens in a bystander manner. Finally, the proteins contained in these exosomes were shown to have a regulatory profile related to nucleic acid binding and enzymes. As mentioned above, sEV not only contain proteins, but they also carry miRNAs that could induce a response in recipient cells, as demonstrated by Bryniarski et al. in 2013 [74]. In their study, sEV-derived CD8+ T cells contained miRNA-150, which acted on macrophages, thereby suppressing delayed-type hypersensitivity (DTH), as shown in an experimental murine model. The action of sEV derived from activated CD8+ T cells has also been demonstrated by Seo et al. [75], where they show that murine sEV disrupts tumor progression by specifically targeting lesional mesenchymal cells. These sEV contain key enzymes such as granzyme and perforin, which are responsible for inducing direct cytotoxicity against tumor cells, thus demonstrating the therapeutic potential of CD8+ T cell-derived sEV in tumor treatment. Most recently, in 2021, Schneider et al. [76] demonstrated the importance of CD73 produced by human CD8+ T cell-derived sEV in immune suppression. In detail, the activation of the immune system and inherent processes such as inflammation promoted the release of ATP into the extracellular space. When present at high levels, ATP is rapidly metabolized by enzymes that degrade ATP into adenosine through the activity of CD39 (which dephosphorylates ATP and ADP into AMP) and CD73 (which converts AMP into adenosine). Activation of the adenosine receptor (predominantly expressed on T cells) leads to an increase in intracellular cAMP, resulting in decreased T cell activation and effector function. Considering this data, they first demonstrated that human Tregs do not generate sufficient adenosine to suppress T cell proliferation, whereas CD8+ T cells displayed high expression of CD73. Furthermore, they revealed that CD73 contained in sEV derived from activated CD8+ T cells is sufficient to degrade AMP and dampen the proliferation and function of activated T cells. This intrinsic mechanism of T cells, in concert with the high ATPase activity of Tregs, mediates adenosine production from ATP and ensures sufficient immune suppression. Lastly, the researchers isolated sEV from the synovial fluid of patients with juvenile idiopathic arthritis (JIA) and showed that sEV plays a crucial role in suppressing T cells in a CD73-dependent manner, achieved through the degradation of AMP. This study highlights the significance of CD73 in sEV as a key factor in regulating inflammation.

### 4.2. CD4+ T Cells

Like CD8+ T cells, CD4+ T cells are also generated in the thymus. Unlike CD8+ T cells, which directly kill infected and tumoral cells, CD4+ T cells secrete cytokines and express surface molecules that interact with other immune molecules on cells. The CD4+ T cell compartment is constituted by distinct subsets of CD4+ T helper (Th) cells described around 37 years ago [77]. Recognition of antigens presented by MHC-II in the presence of costimulatory signals results in cellular activation, proliferation, and differentiation into distinct subsets of effector Th cells [70,78]. The subsets include Th1, Th2, Th9, Th17, Th22, T follicular helper (Tfh), and T regulatory cells (Tregs) [79]. The differentiation of each type depends on the cytokine environment present during activation. [78]. Regarding vesicle production, it was first reported in 1999 that Jurkat cells (human) release microvesicles upon PHA stimulation, which bear FasL and APO2 ligand, two molecules involved in cell death; therefore, the association between microvesicles release by activated CD4+ T cells was centered primarily on apoptosis [80]. Then, the group led by Hivroz et al. reported that, indeed, CD4+ T cells release sEV only if activation is provided via TCR. In their work, the authors comprehensively characterized the vesicles, which had a size between 50 and 100 nm, displayed a round shape and harbored the molecules CD63, CD2, LFA-1, and CXCR4. It is important to highlight that in this report it is demonstrated that CD4+ T cell-derived sEV do not correspond to apoptotic blebs [81]. In a mouse model using an antigen-specific system (OVA-pulsed DCs and OVA-TCR transgenic CD4+ T cells, or OT-II), it was shown that OT-II-derived sEV can be uptaken by DCs in an MHC-II and LFA-1 specific manner since the addition of blocking antibodies against these molecules prevented sEV capture by DCs. Following experiments, it was demonstrated that these OT-II-derived sEV inhibit CD4+ T cell proliferation in vitro and the cytotoxic activity of CD8+ T cells in vivo using the OVA-expressing B16 melanoma model. Based on these observations, the authors proposed the use of antigen-specific CD4+ T cell-derived sEV as an immunosuppressive tool [82]. Considering that activated CD4+ T cells play a role in atherosclerosis by infiltrating atherosclerotic plaques, Zakharova and colleagues investigated whether sEV derived from human CD4+ T cells may affect cholesterol production/distribution on monocytes. Human-activated CD4+ T cells released sEV that induced cholesterol accumulation and TNF-α production on monocytes, suggesting a detrimental effect of sEV in the atherosclerosis setting [83]. In line with these observations, another study demonstrated that activated CD4+ T cell-derived sEV induced the differentiation, proliferation, and migration of cardiac fibroblasts in vitro. Using a murine myocardial infarction (MI) model, the authors found that the administration of activated CD4+ T cell-derived sEV elicited a deterioration of cardiac function post-MI, which was dependent on miR-142-3p, a miRNA enriched in the plasma of heart transplant patients [84,85]. Finally, in a clinical setting of chronic liver diseases, a study reported that CD4+ T cell-derived sEV isolated from the blood of active chronic hepatitis C (CHC) patients is more abundant than those obtained from non-alcoholic fat liver (NAFL) disease or from nonalcoholic steatohepatitis (NASH), suggesting the use of sEV quantity to distinguish between these diseases’ activity [86].

### 4.3. T Regulatory Cells

T regulatory cells (Tregs) are a subset of CD4+ T cells considered a regulator of the immune response that maintains self-tolerance and immune homeostasis by eliminating autoreactive T cells, inducing self-tolerance, curbing inflammatory processes, and inhibiting the function of effector T cells, B cells, and other leukocyte populations. These cells are characterized by high expression of CD25 (IL-2 receptor alpha chain) and specific expression of the transcription factor Forkhead box protein P3 (FoxP3). The development of Tregs occurs in the thymus (giving rise to natural Tregs, nTreg), but can also take place in the periphery (peripheral Tregs, pTregs) [87,88]. Tregs can suppress immune responses through different mechanisms, like the expression of co-inhibitory surface molecules, the production of anti-inflammatory metabolites, the secretion of cytokines, and the release of sEV with suppressive activity. [89,90,91]. In 2013, Smyth et al. reported that murine Tregs secrete sEV following TCR activation [92]. These sEV suppress CD4+ T cell proliferation and IFN-γ secretion. Interestingly, the presence of the molecule CD73 on sEV is essential to suppressing the immune response. In detail, the authors show that sEV from Tregs contains markers such as CD4, CD2, MHC-I, CD25, CTLA-4, and CD73. Interestingly, the suppressive activity of Tregs-derived sEV did not depend on CTLA-4 presence on the sEV since neutralizing antibodies for CTLA-4 did not affect sEV regulatory function. However, sEV obtained from a CD73-deficient CD4+ T cell line did not show suppressive activity, demonstrating the relevant function of CD73 on Tregs-derived sEV. In 2014, Okoye et al. [93] demonstrated that murine Tregs produced the highest concentration of sEV among other lymphocytes and that Tregs-derived sEV contained premature and mature miRNA, which are necessary to inhibit Th1 cell function. In detail, Tregs were transfected with fluorescent oligonucleotide duplexes (FL-dsRNA) to visualize miRNA transfer into target cells. The authors then identified miR-155, Let-7b, and Let-7d (mature miRNAs) and one pre-miRNA, Hp_miR-344d-2, as the molecules involved in the suppression of T cell proliferation and Let-7d as the main miRNA responsible for the inhibition of Th1 function in vivo. By using elegant approaches, the authors demonstrated that miRNA transfer via sEV is required by Tregs to exert their regulatory effect on Th1 cells. Last year, our group demonstrated that murine Tregs release sEV that contains Neuropilin-1 (Nrp1), a protein necessary to block transplant rejection, as shown in a murine skin transplant [89]. This study demonstrated in vivo that wild-type Tregs-derived sEV exerted a suppressive function, decreasing proliferation and cell division of polyclonally activated conventional T cells, while Nrp1KO Tregs-derived sEV had an altered suppressive function, suggesting that the presence of Nrp1 in sEV is involved in immune function. In vivo, these Nrp1KO Tregs-derived sEV are deficient in inducing immune tolerance by affecting the presence of anti-inflammatory macrophages (M2) in grafted tissue.

In vitro, Tregs can be induced from CD4+FoxP3- T cells (and from naïve CD4+ T cells) by different protocols. The best-known procedure is the use of high concentrations of IL-2 in addition to TGF-β1, in which the resulting Treg population is named “induced” Tregs, or iTregs. In addition, other protocols include IL-2, TGF-β1, and all-*trans*-retinoic acid (ATRA), a procedure that generates Tregs with a very stable phenotype [94]. The observation of iTregs-derived sEV was made by Chen et al., demonstrating their suppressive role in a mouse model of collagen-induced arthritis (CIA). In detail, they first induced iTregs in vitro, isolated their sEV, and showed the presence of the canonical sEV markers CD9, CD63, CD81, and TSG101. Using a suppression assay in vitro, they demonstrated that iTregs-derived sEV exerts comparable regulatory activity as sEV obtained from nTregs. This finding was observed in vivo, as mice subjected to CIA and treated with iTregs-derived sEV showed a decreased Th17/Tregs ratio. These effects were mediated by miR-449a-5p via Notch signaling [95].

### 4.4. T Follicular Helpers

**Figure 3:**
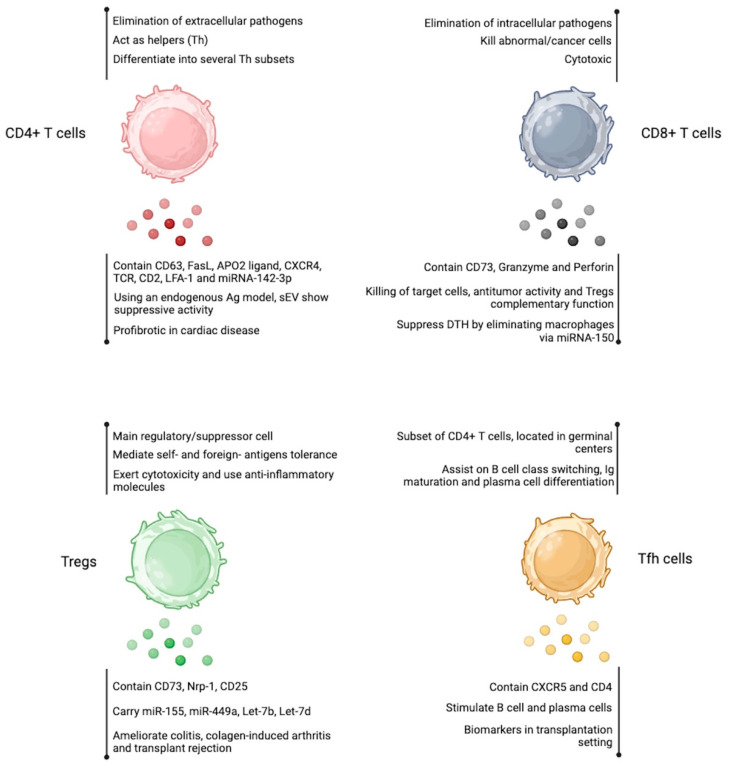
Extracellular vesicles produced by T cell-subsets. EV obtained from CD4+ T cells, CD8+ T cells, Tregs, and Tfh cells resembles the properties of the cell of origin. The detailed effects are shown below each cell cartoon. Figure created at BioRender.com.

Follicular helper T cells (Tfh) are a subset of CD4+ T cells that play a crucial role in orchestrating humoral immunity [96]. They have a characteristic phenotype that includes the expression of surface markers such as CXC chemokine receptor 5 (CXCR5), programmed cell death protein 1 (PD1), inducible T cell costimulatory (ICOS), and the transcription factor Bcl-6 [97]. Mature Tfh cells are in the follicles of secondary lymphoid organs, where they assist B cells in the germinal centers to facilitate immunoglobulin affinity maturation, class switch recombination, and the generation of plasma cells from long-lived and memory B cells [97,98]. Tfh cells are also essential in immune responses to the influenza vaccine, where they seem to regulate antibody production and B cell memory formation [99]. On the other hand, an exaggerated response and an increased number of Tfh cells can lead to autoimmune diseases [100]. There are only a couple of reports describing the capacity of human Tfh cells to secrete sEV in the transplantation setting. It has been reported that human Tfh cells are closely related to the onset and development of antibody-mediated rejection (AMR) after kidney transplantation [101]. In fact, Yang et al. reported that renal transplantation patients have CD4+ CXCR5+ sEV in peripheral blood, suggesting their Tfh cell origin. In vitro, the authors showed that sEV from AMR patients stimulates B cells and plasma cells, suggesting crosstalk between Tfh cell-derived sEV and B cells as a response to renal transplantation [102]. Figure 3 summarizes the characteristics and functions of CD8+ T cells, CD4+ T cells, Tregs, and Tfh cell-derived sEV.

## 5. Conclusions

Overall, all immune cells secrete sEV, and, in some cases, one could induce an anti-inflammatory or a pro-inflammatory phenotype, depending on the disease that needs to be treated. This means that, in theory, most of the diseases in which inflammation has to be enhanced or inhibited (for example, solid tumors or autoimmunity, respectively) could be treated by the administration of sEV. Because of this undeniable versatility, sEV is an attractive tool to explore in the clinical setting. This opportunity deserves its own investigation (basic and clinical research) since the potential administration of sEV instead of cells could bypass cell plasticity. In other words, the use of cells brings as a possibility a potential change in their phenotype/function once administered, whereas sEV should not suffer any variations regarding the inflammatory milieu they encounter in the patient. Aspects such as the production of sufficient numbers of sEV required per patient or strategies to efficiently target sEV to a cell type or tissues are other considerations to take into account in the design of sEV therapy, which are currently under study to advance in the biomedical field.

## Data Availability

Not applicable.
